# A Rare Case of Penile Cutaneous Horn With Underlying Well-Differentiated Squamous Cell Carcinoma: A Case Report

**DOI:** 10.7759/cureus.96505

**Published:** 2025-11-10

**Authors:** Varsha Raja Ayyanar, Vasudeo Ridhorkar

**Affiliations:** 1 Department of Urology, Krishna Institute of Medical Sciences (KIMS) - Kingsway Hospitals, Nagpur, IND

**Keywords:** keratinous lesion, partial glansectomy, penile cutaneous horn, rare case report, squamous cell carcinoma

## Abstract

A hard, conical growth that protrudes from the skin's surface and resembles a small horn is known as a cutaneous horn or cornu cutaneum. The horn consists of compact keratin fibers. Cutaneous horns commonly develop on photo-exposed regions and, in exceedingly rare cases, the penis. Although cutaneous horns can develop on healthy skin, they are more widely associated with preexisting skin conditions. We report a rare case of a penile cutaneous horn in a 55-year-old man presenting with pain and a growth on his penis that had persisted for a year. The patient underwent a penile glansectomy under spinal anesthesia. Histopathology revealed a well-differentiated squamous cell carcinoma, indicating malignancy.

## Introduction

The condition known as penile horn, or cornu humanum, refers to a hyperkeratotic lesion found on the glans penis. The compact keratin that makes up the horn results from several epidermal modifications. This phrase was used to describe lesions in which the height of the keratitis mass exceeds half of its diameter. While cutaneous horns are commonly found on photo-exposed areas like the face and scalp, they can also develop on other parts of the body, including the hands, eyelids, nose, chest, neck, shoulder, and, in extremely uncommon cases, the penis [1]. These horns occur more frequently on preexisting skin disorders such as warts, keratosis, nevi, trauma, burns, lupus vulgaris, and even epithelioma than on normal skin [2]. Since the first instance, documented in 1854, the incidence of penile cutaneous horn has been exceptionally low. Only 30 of these occurrences have been documented in the literature during a 25-year period, which is a small number [2]. We report a rare case of penile cutaneous horn in a 55-year-old man in which the histological diagnosis at the base of the horn after a penile glansectomy was a well-differentiated squamous cell carcinoma.

## Case presentation

**Figure 1:**
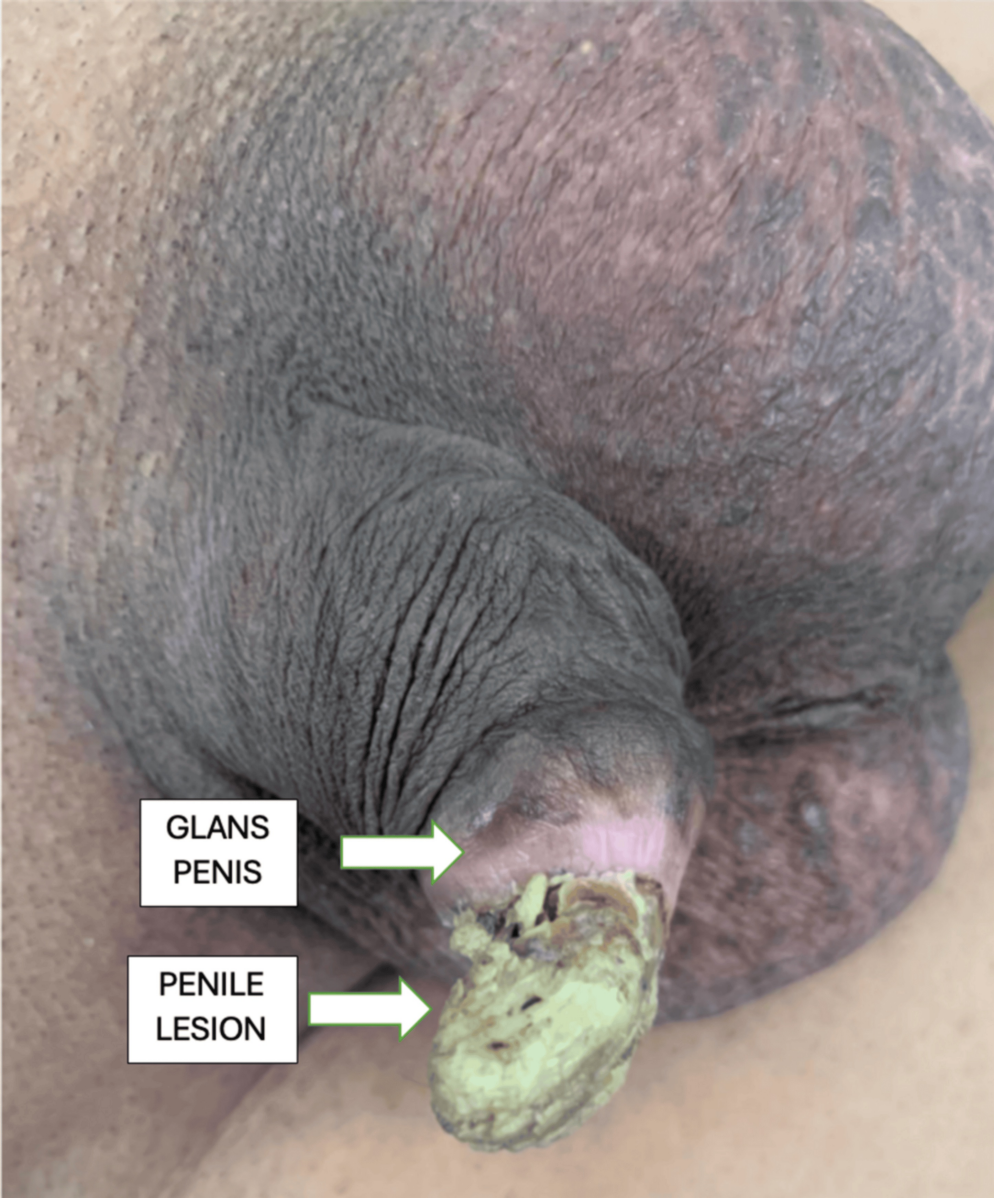
Lesion from the left lateral aspect

**Figure 2:**
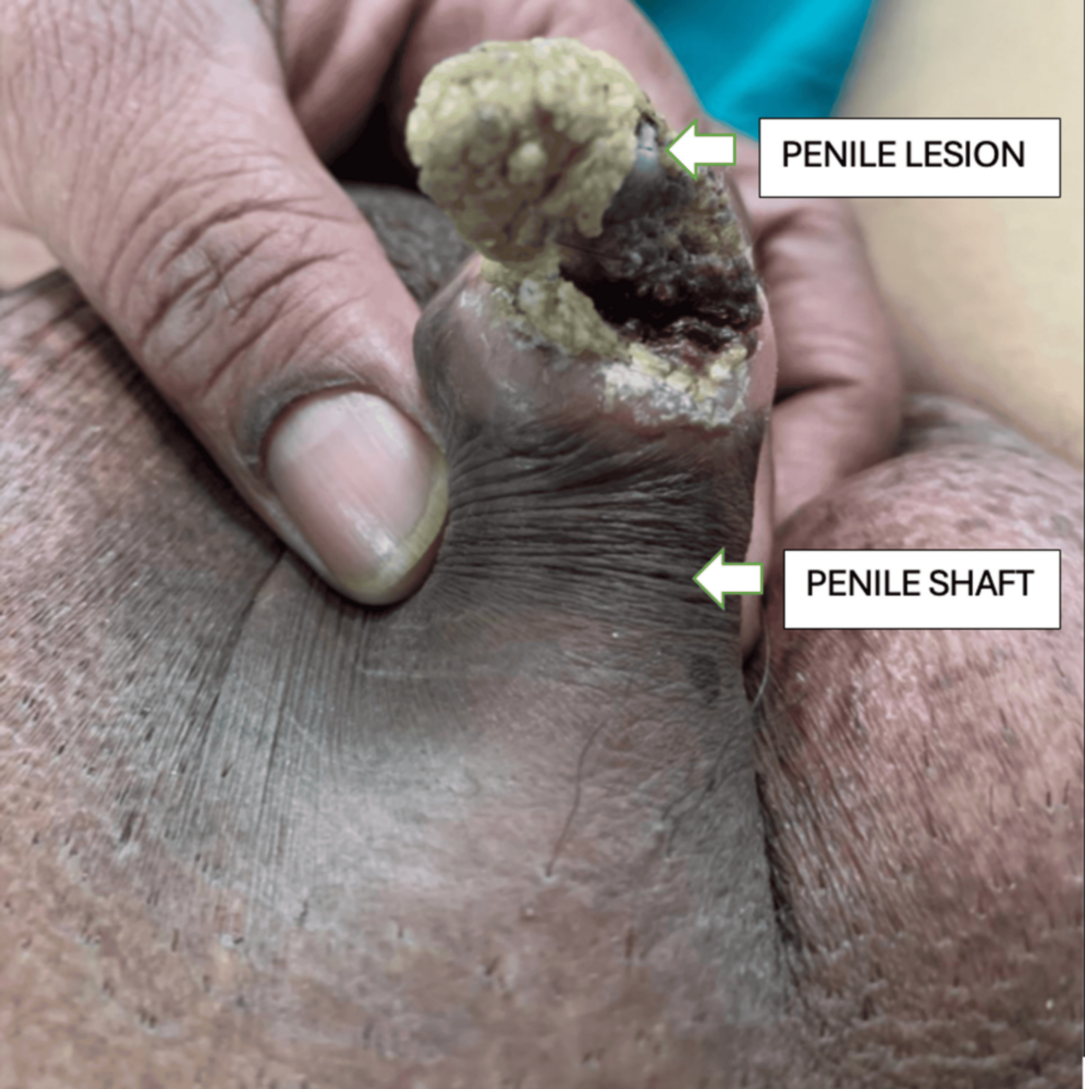
Lesion from the inferior aspect

A 55-year-old man visited the hospital with pain and a hard growth in his penis that had been bothering him for a year. The growth was initially less than 5 mm and asymptomatic. Later, it increased in size, approximately 3 cm, over a period of six years, causing discomfort. There was no history of penile urethral discharge, bleeding from the growth, fever, or weight loss. The patient has been married for 30 years and has four children. His past medical history includes hypertension and epilepsy, for which he takes regular medications. On local examination, there was a cone-shaped yellowish lesion (Figures 1, 2) on the ventral surface of the glans penis with a wide base approximately 2-3 cm, nontender. The skin surrounding the lesion appeared normal, with palpable induration over the entire glans, and no palpable inguinal lymphadenopathy was noted. In compliance with ethical standards, the patient provided informed consent for the use of their preoperative and postoperative photographs in this educational material.

Surgical procedure

**Figure 3:**
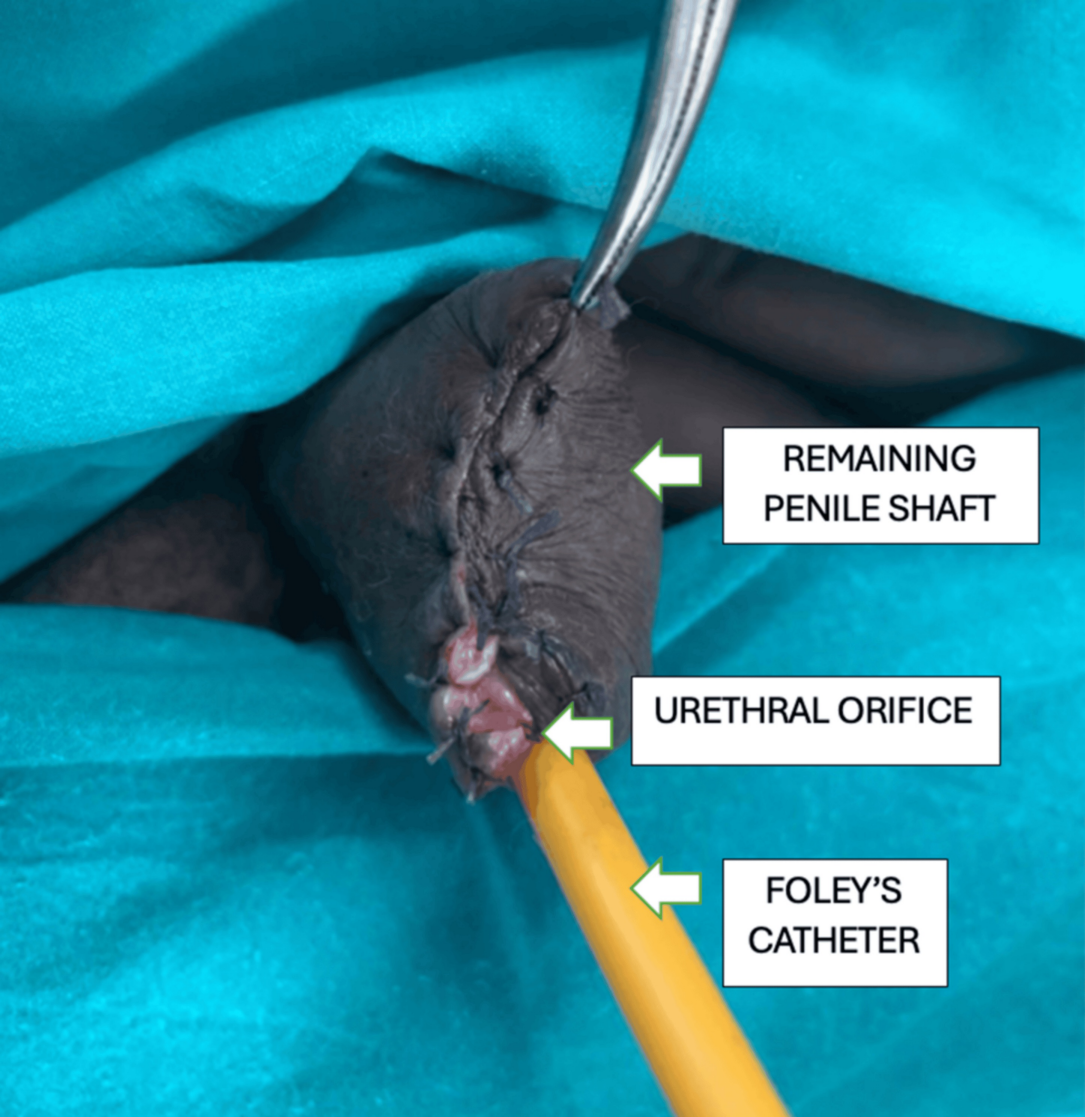
Image after a partial amputation of the penis with a Foley catheter in situ

**Figure 4:**
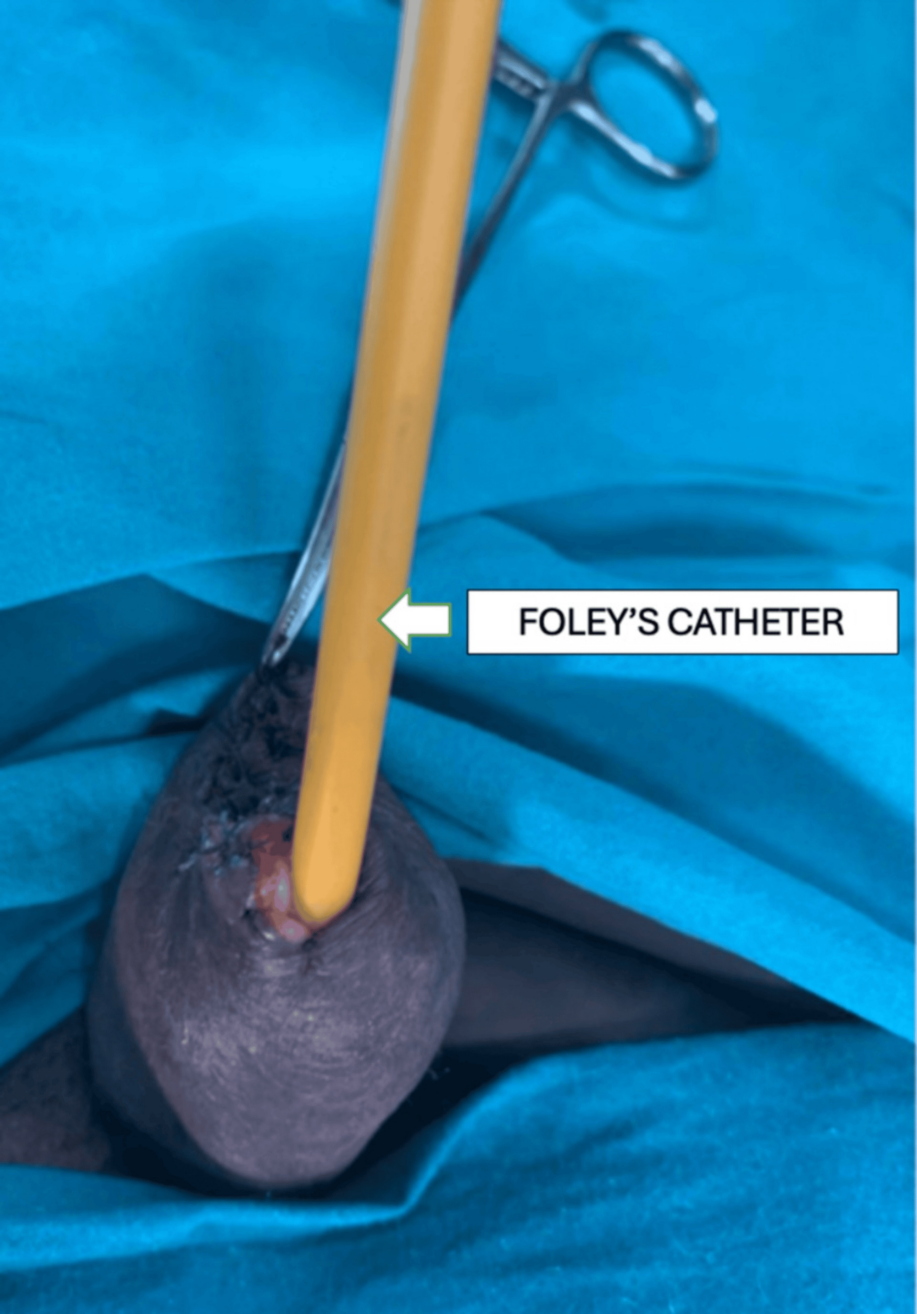
Foley's catheter in situ after surgery

The preoperative diagnosis was penile cutaneous horn. The patient was counseled for penile glansectomy, a partial amputation of the penis. Under spinal anesthesia, an elective partial amputation was performed in the supine position. A circumcoronal incision was made, ensuring adequate clearance and margin. The urethra was dissected and looped up. The corporal division was performed, and hemostatic sutures were placed using absorbable material. The urethra was kept longer than the corporal cut margins, and hemostasis was confirmed. The urethra was sutured to the corpora with interrupted fixation sutures. The fascia was closed over the corpora, and the skin to the urethra was sutured using absorbable sutures (Figure 3). A 16-F Foley catheter was inserted for bladder drainage (Figures 3, 4).

Histopathology

**Figure 5:**
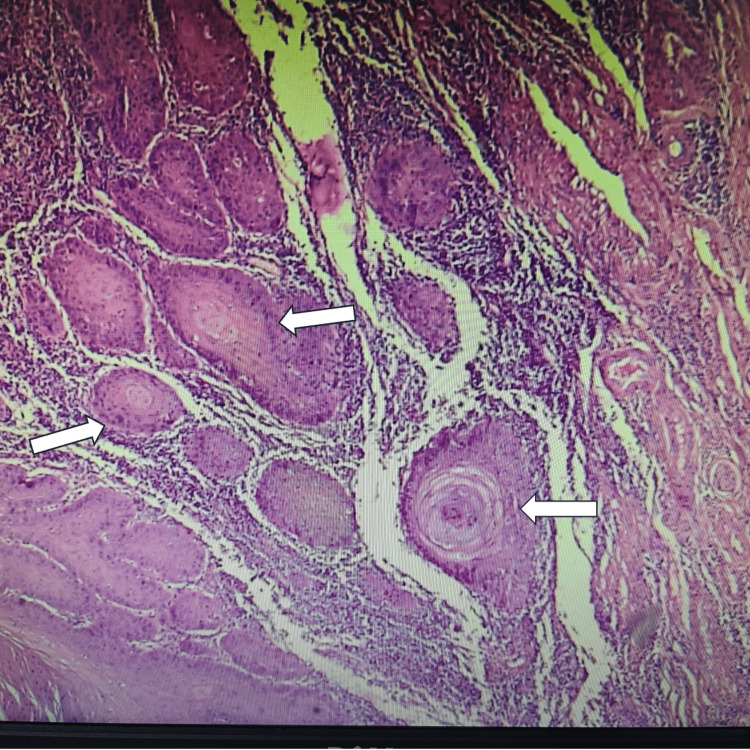
Histopathological image postoperatively showing prominent keratin pearls (white arrows) within tumor nests, a hallmark of squamous differentiation

**Figure 6:**
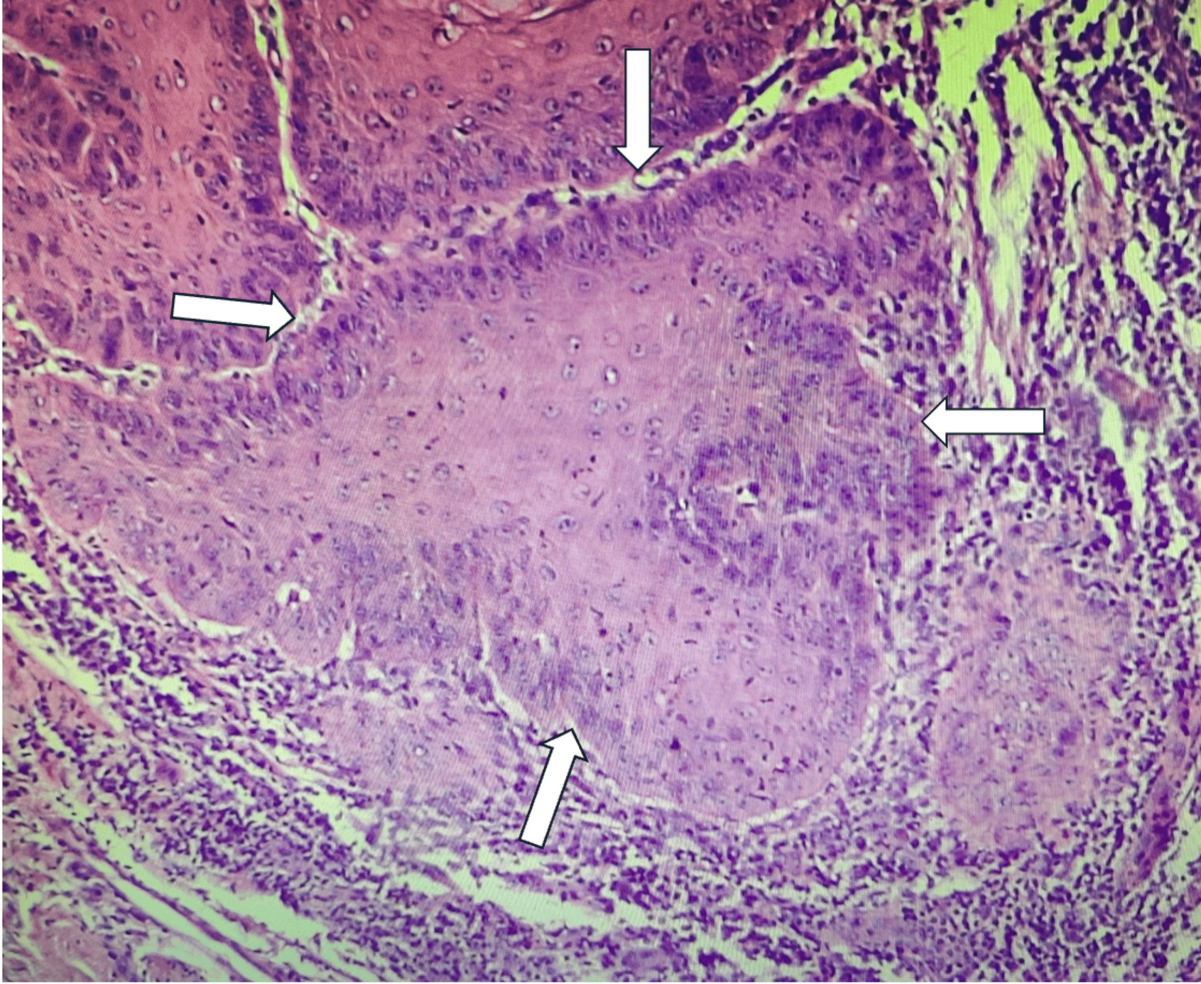
Larger irregular nests of atypical squamous cells with disorganized architecture. Individual cell keratinization and dyskeratosis are evident (white arrows)

**Figure 7:**
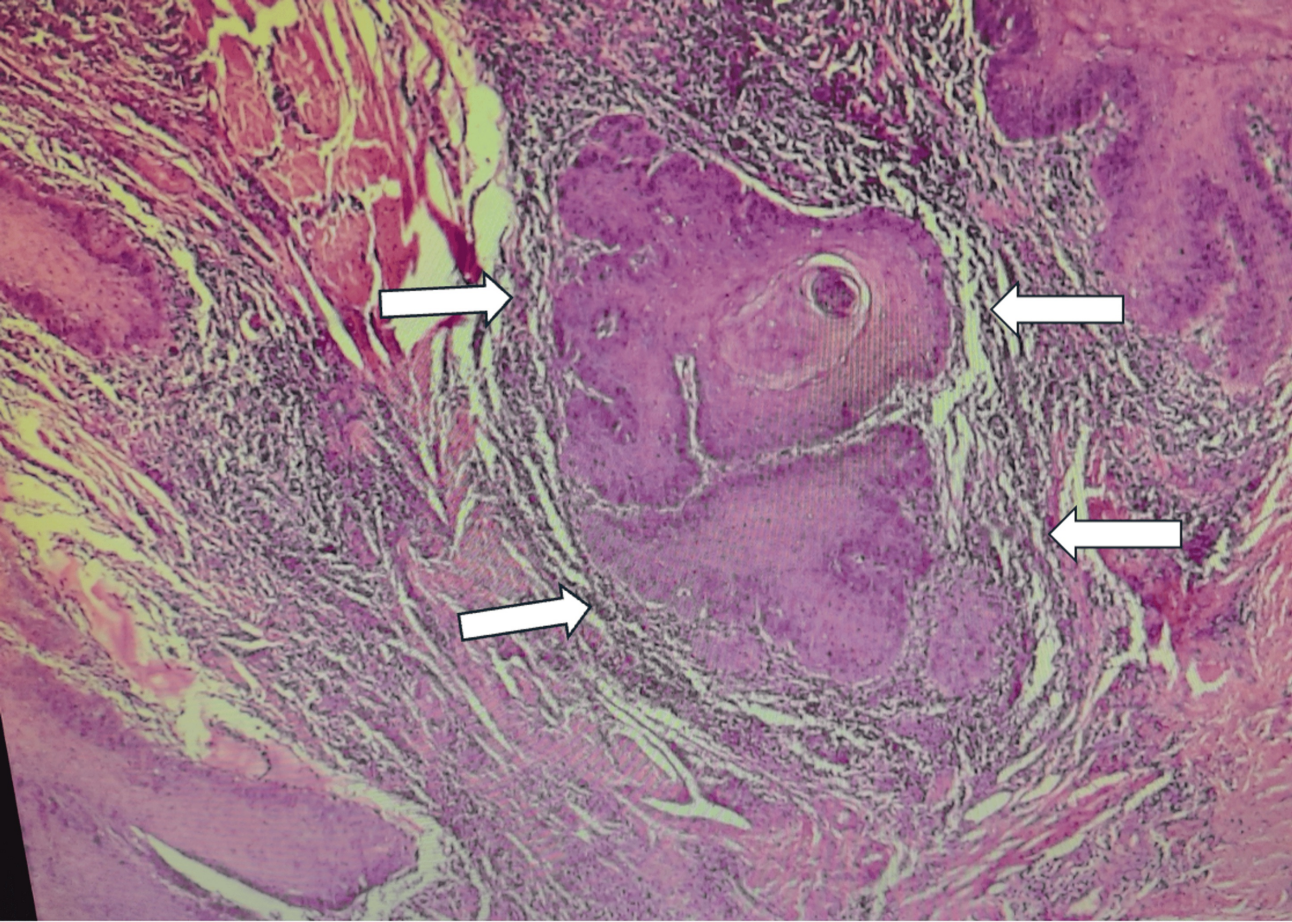
Nests and sheets of malignant squamous epithelium deeply infiltrating fibrous stroma. Clear keratin pearl formation and concentric lamellation of keratin (white arrows); features consistent with well-differentiated squamous cell carcinoma

The amputated penis with the horn was sent for histopathological analysis. Postoperatively, the patient recovered satisfactorily with no complications. He was followed up in the clinic in two weeks postoperatively and then after six months. Gross examination revealed a glans penis with lesions measuring approximately 3 × 2 × 1.5 cm. The horn-like structure measured 2.5 × 2 × 1 cm and was 0.4 cm away from the closest surgical margin. Multiple deeper tissue pieces, each measuring 1 × 1 × 0.6 cm, were also examined. Microscopy revealed a tumor with both exophytic and endophytic components. The exophytic part demonstrated marked hyperkeratosis with keratin pearls (Figure 5), and the endophytic component showed an infiltrative tumor with mild-to-moderate nuclear atypia, suggestive of a well-differentiated squamous cell carcinoma (Figures 6, 7). No lymph-vascular or perineural invasion was observed.

## Discussion

A conical, hyperkeratotic protrusion known as a cutaneous horn frequently resembles an animal horn. The term “cutaneous horn” is a clinical descriptor rather than a diagnosis. Over 150 cases have been documented in the literature [1]. The first instance of cutaneous horn, or cornu cutaneum, was recorded in 1588 in an elderly Welsh woman, whereas the first penile cutaneous horn case report was published in 1854 [2]. Most cases occur in men over 50 years of age. They present as keratinous, white, or yellowish protrusions extending from the glans penis. These lesions may range from millimeters to centimeters and are often nontender and asymptomatic. The exact cause of penile horns is unclear, but they may result from compacted keratin formed by dried sebaceous cyst secretions over time [3].

Even when initial histology shows benign lesions, penile cutaneous horns may recur or transform malignantly on repeat biopsy [4]. As stated by Lowe and McCullough [5], cutaneous horns may be benign (42%-56%), premalignant (22%-37%), or malignant (20%-22%); however, the European Association of Urology guidelines state that penile cutaneous horns are premalignant lesions [2]. It has been suggested that penile carcinogenesis follows a bimodal pathway, one involving human papillomavirus (HPV) and the other a nonviral pathway, such as chronic preputial irritation, phimosis, surgical trauma (including circumcision) [6], condyloma acuminatum [7], and radiotherapy [8]. Verrucous carcinoma, a well-differentiated variant of squamous cell carcinoma, is linked to smoking, HPV, and chronic inflammation [9]. In our case, it is impossible to know the temporal relationship between any former lesion and the development of the penile horn.

Multiple studies have shown that p16^INK4A ^immunohistochemical expression serves not only as a marker of high-risk HPV infection but also aids in distinguishing penile epithelial abnormalities and precancerous lesions [10]. DNA polymerase chain reaction can also be used to diagnose HPV infection, as these infections lead to penile carcinogenesis.

Management of penile cutaneous horn primarily involves complete surgical excision of the lesion with a representative biopsy obtained from its base. Histopathological examination of this biopsy is essential, as it determines the underlying cause, which may range from benign dermatological conditions to premalignant or malignant neoplasms. While surgery remains the gold standard [11], alternative treatment approaches have also been described, including laser therapy and the application of keratolytic agents. Carbon dioxide and neodymium: yttrium-aluminum-garnet laser techniques are especially useful because they produce minimal scarring and provide superior cosmetic results, which is particularly important given the sensitive anatomical site [12].

In situations where malignancy is confirmed, partial penectomy is generally advised to achieve oncological clearance while preserving as much penile tissue and function as possible, with or without regional lymph node dissection. Imaging studies can also aid in management; magnetic resonance imaging (MRI), for instance, is particularly valuable when there is diagnostic uncertainty about tissue infiltration [13]. Preoperative MRI helps assess the depth and extent of invasion, thereby informing surgical planning, facilitating patient counseling, and assisting in predicting both functional and cosmetic outcomes. Regular follow-up is recommended, as recurrence or progression can occur depending on the underlying histology and adequacy of initial treatment.

## Conclusions

Penile cutaneous horn is a rare hyperkeratotic lesion often associated with underlying premalignant or malignant pathology; this case is one of the few histologically confirmed cases of penile horn reported in the literature. This case report also highlights the need to consider squamous cell carcinoma in such presentations, especially in older men. The patient has been regularly followed up every six months since then and has been disease-free. Prompt surgical excision and histopathological examination are essential for diagnosis and management. Regular follow-up is recommended to monitor recurrence and malignant transformation.
